# Comparison of surgical outcomes of osteosynthesis using anatomical locking plates with proximal screws and smooth pegs for proximal humeral fractures

**DOI:** 10.1186/s12891-025-08917-0

**Published:** 2025-07-09

**Authors:** Ryogo Furuhata, Atsushi Tanji, Yusaku Kamata, Noboru Matsumura

**Affiliations:** 1 Department of Orthopaedic Surgery, Ashikaga Red Cross Hospital, 284-1 Yobe-cho, Ashikaga-shi, Tochigi 326-0843 Japan; 2 Department of Orthopaedic Surgery, National Hospital Organization Tokyo Medical Center, Meguro-ku, Tokyo, Japan; 3 Department of Orthopaedic Surgery, Keio University School of Medicine, Shinjuku-ku, Tokyo, Japan

**Keywords:** Proximal humeral fracture, Plate fixation, Outcome, Peg, Greater tuberosity reduction loss

## Abstract

**Background:**

Plate fixation is a widely used surgical procedure for proximal humeral fracture; however, relatively high complication rates have been reported. To prevent the occurrence of proximal screw cut out, pegs with blunt tips have been introduced to replace traditional screws for fixation of the humeral head. However, few reports are available on the effect of using smooth pegs on surgical outcomes. In this study, we aimed to compare the postoperative outcomes of osteosynthesis using anatomical plates using all proximal screws and using all proximal pegs.

**Methods:**

We retrospectively identified 48 patients who underwent osteosynthesis using an anatomical locking plate for proximal humeral fractures. We divided the patients into a screw group (25 patients) and a peg group (23 patients) according to the devices used to fix the humeral head. We compared operative outcomes, postoperative shoulder functional scores, and postoperative complication rates between the two groups.

**Results:**

In terms of operative outcomes, the operation time was significantly shorter in the peg group than that in the screw group (94 [83–105] min vs. 114 [98–124] min, *p* < 0.001, *r* = 0.52). No significant differences in adjusted Constant score and American Shoulder and Elbow Surgeon score were observed at 1 year postoperatively between the two groups. However, the incidence of greater tuberosity reduction loss was significantly higher in the peg group than that in the screw group (17.4% vs. 0%, *p* = 0.046, φ = 0.32).

**Conclusions:**

Our study showed that the use of proximal pegs did not significantly affect postoperative shoulder functional outcomes, although it may reduce operative time. In three-part fractures involving the greater tuberosity, the use of proximal screws or plates with sufficient coverage of the greater tuberosity may be desirable to prevent reduction loss of the greater tuberosity.

**Supplementary Information:**

The online version contains supplementary material available at 10.1186/s12891-025-08917-0.

## Background

Proximal humeral fractures are the third most common fractures in adults [1, 2]. Surgical procedures for displaced proximal humeral fractures include osteosynthesis using an intramedullary nail or a locking plate, hemiarthroplasty and reverse shoulder arthroplasty [3]. Plate fixation is a widely used surgical procedure that produces satisfactory surgical outcomes [4, 5]. However, plate fixation is associated with a relatively high frequency of postoperative complications, ranging from 33 to 42% [6–8]. In particular, proximal screw perforation is the most common complication [6, 7] and the reason for revision surgery [9]. This complication is related to multiple risk factors, including older age [7], female sex [7], poor bone quality [10], complex fracture type [7, 11, 12], malreduction (postoperative varus deformity) [12], absence of calcar screws [12], and lack of metaphyseal support [12]. To prevent penetration of the implant into the glenohumeral joint, dual plate fixation [11, 12], medial support screws [13–15], and the use of fibular strut grafts [15] or calcium phosphate cement [15, 16] have been attempted. In addition to these various attempts, pegs with blunt tips are introduced to replace traditional screws for fixation of the humeral head. The use of pegs also has the potential to reduce the damage to the glenoid surface even if penetration occurs [17].

Previous cadaveric biomechanical studies have reported that plates with smooth pegs exhibit biomechanical characteristics equivalent to those with threaded screws in proximal humeral fracture models [18, 19]; however, a finite element study reported that threading the smooth peg increased the varus bending stiffness of proximal humeral plate constructs [20]. Although recent advances have improved understanding of the biomechanical characteristics of plate fixation using pegs, the superiority of the fixation strength of pegs versus screws remains controversial.

In Japan, the ALPS Proximal Humerus Plating System (Zimmer Biomet, Warsaw, IN, USA) was introduced in 2023, enabling smooth pegs to be used to fix the humeral head. However, few reports are available on the effects of using smooth pegs on surgical outcomes [21–23].

Therefore, the purpose of this study was to compare the functional and radiological outcomes after osteosynthesis of proximal humeral fractures using anatomical plates using all proximal screws and using all proximal pegs.

## Methods

### Study design and patients

**Fig. 1:**
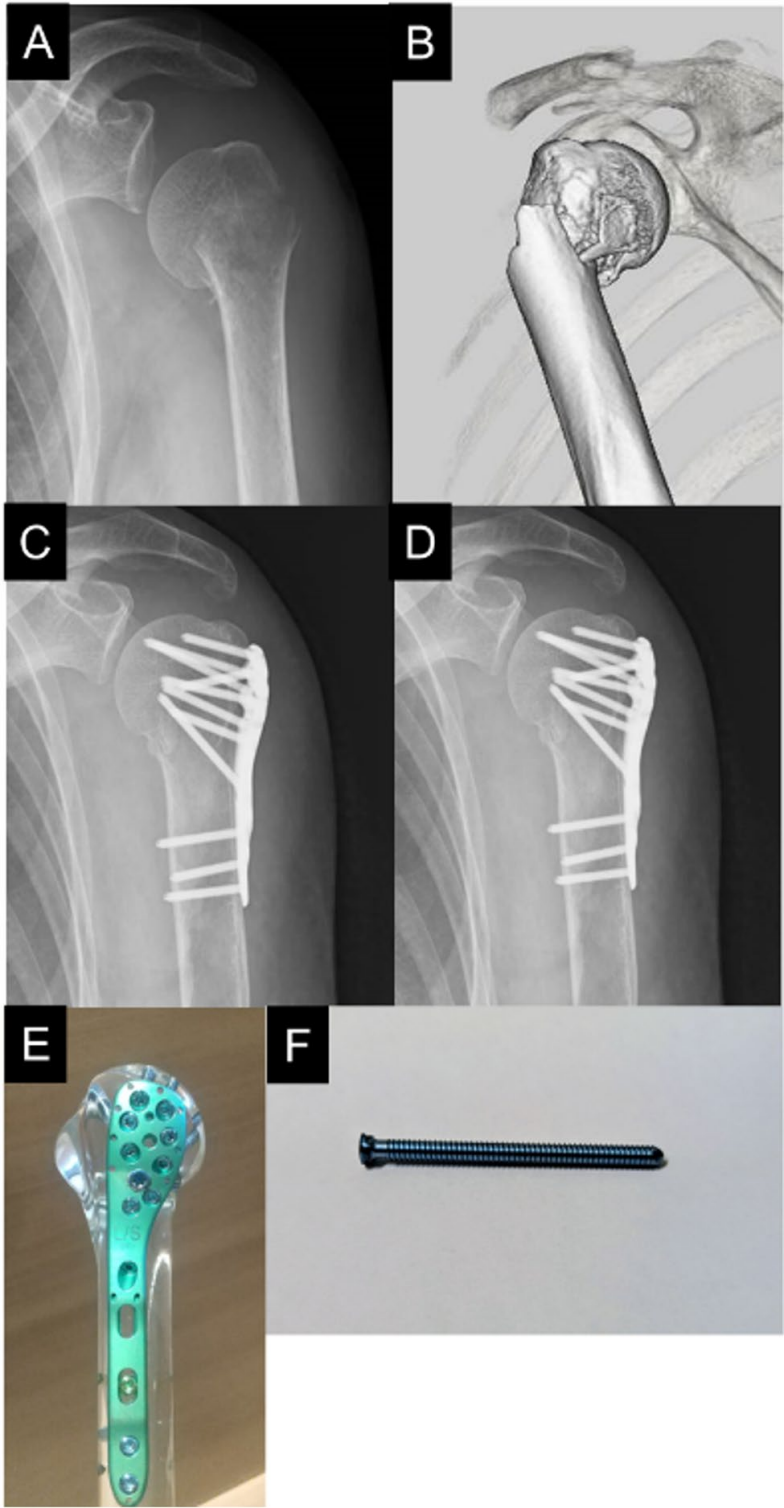
Clinical images and photographs of the plate fixation using MODE^®^ plate with all screws. Preoperative radiograph (**A**), three-dimentional compuded tomography image (**B**), and radiograph immediately after surgery (**C**) and 1 year at postoperatively (**D**) of the patient who underwent plate fixation using MODE^®^ plate with all screws. Photograph of MODE^®^ plate (**E**) and proximal screw (**F**)

**Fig. 2:**
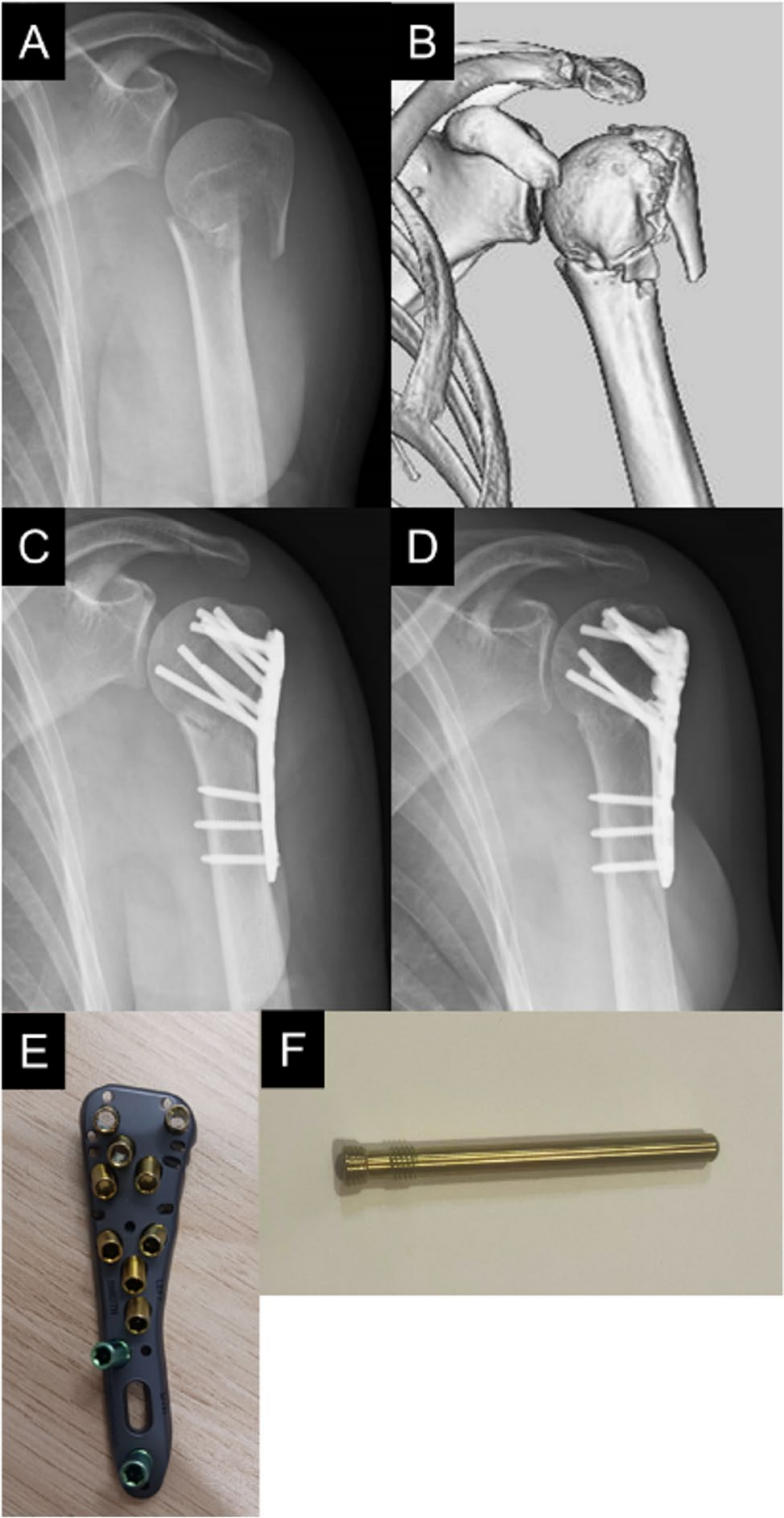
Clinical images and photographs of the plate fixation using ALPS plate with all screws. Preoperative radiograph (**A**), three-dimentional compuded tomography image (**B**), and radiograph immediately after surgery (**C**) and 1 year at postoperatively (**D**) of the patient who underwent plate fixation using ALPS plate with all screws. Photograph of ALPS plate (**E**) and proximal screw (**F**)

The study protocol was approved by the independent ethics committee of our hospitals. This is a retrospective study of patients who underwent plate fixation for proximal humeral fractures performed at two general hospitals during the period 2018–2019 and 2022–2023. We excluded the coronavirus disease 2019 epidemic period (2020–2021) because insufficient postoperative care and rehabilitation due to hospital visit restrictions could have affected the postoperative outcomes. During this study period, we performed plate fixation in all patients with two-part or three-part (Neer classification [24]) proximal humeral fractures except those at high risk of postoperative humeral head necrosis (posteromedial metaphyseal head extension, disruption of medial hinge) [25]. In our hospitals, we used the MODE^®^ proximal humeral plate (MDM, Tokyo, Japan) with all proximal screws before March 2023 (Fig. 1) and the high plate of the ALPS plate with all proximal pegs after March 2023 (Fig. 2).

In this study, we included patients (≥ 18 years) who underwent plate fixation for proximal humeral fractures during this period and were followed-up for 1 year postoperatively. The exclusion criteria were Neer type I, IV-VI fractures, high risk of postoperative humeral head necrosis (posteromedial metaphyseal head extension, disruption of the medial hinge), paralysis of the affected upper extremity due to cerebral infarction or other causes, multiple injuries (multiple fractures or concomitant organ injury), and previous surgery on the involved shoulder. We categorized the patients who underwent plate fixation using the MODE^®^ plate with all proximal screws by March 2023 into the screw group and those who underwent plate fixation using the ALPS plate with all proximal pegs after March 2023 into the peg group.

### Surgical procedure

One orthopedic surgeon with over 10 years of experience in shoulder surgery performed surgery under general anesthesia with the patient in the beach-chair position. The surgery was performed with one assistant. A total of 14 assistants with 1–3 years of experience in trauma surgery were involved in this study. A delto-pectoral approach was used in all cases. We reduced the fracture fragments using two No. 2 Fiver wires (Arthrex, Naples, FL, USA) attached to the rotator cuff muscles. We reduced the fracture fragments until “anatomical” or “acceptable” reduction status, as reported by Schnetzke et al. [26], was obtained on intraoperative fluoroscopy. In the MODE^®^ plate, the proximal fragment was fixed with screws, whereas, in the ALPS plate, the proximal fragment was fixed with pegs. The screw has a core diameter of 2.9 mm, an insertion angle of 24° (most proximal screw), and is tightened using a torque driver. The peg has a core diameter of 3.2 mm, an insertion angle of 30° (most proximal peg), and is tightened using a torque driver. We first inserted a distal cortical screw. Subsequently, we inserted the proximal screws/pegs in order from the proximal side. Finally, we inserted two distal locking screws. We verified the length of the screws/pegs using intraoperative fluoroscopy (OEC elite, GE health care, Chicago, IL, USA). If the articular cortex in the insertion direction was defective, we did not insert the proximal screws/pegs. We inserted proximal screw/pegs into all holes in which a solid bone cortex could be confirmed in the insertion direction. After plate fixation, we fastened Fiber wires into the plate holes. We did not perform any additional procedures such as screw cement augmentation, bony allograft augmentation, or placement of additional calcar screws or blades.

All patients underwent the following rehabilitation protocol regardless of preoperative fracture type or postoperative reduction status. The patients began passive range of motion training on postoperative day 3 and active range of motion training 4 weeks after surgery. The rotator cuff exercise was initiated 8 weeks postoperatively. Outpatient rehabilitation was performed for 5 months after surgery.

### Outcome measures

We retrospectively evaluated operative outcomes based on the operative time and the amount of bleeding. In this study, we defined the operative time as the duration from the first incision to closure.

Additionally, we retrospectively evaluated reduction status [26] immediately after surgery, postoperative shoulder functional outcomes using the Constant score [27] and the American Shoulder and Elbow Surgeon (ASES) Score [28], visual analogue scale (VAS) at 1 year after surgery, and the postoperative complication (nonunion, screw/peg penetration, avascular necrosis, varus progression, and greater tuberosity reduction loss) rate within 1 year after surgery. Based on the classification by Schnetzke et al. [16], the reduction status is defined as follows: head-shaft displacement > 5 mm, varus head-shaft alignment > 150° or < 110°, or cranialization of the greater tuberosity > 5 mm is considered “malreduced,” while head-shaft displacement ≤ 5 mm, 110° ≤ varus head-shaft alignment ≤ 150°, and cranialization of the greater tuberosity ≤ 5 mm are considered “acceptable.” Among the “acceptable” cases, anatomical head-shaft displacement (< 2 mm), 120° ≤ varus head-shaft alignment ≤ 150°, and anatomical cranialization of the greater tuberosity (< 2 mm) are defined as “anatomical.” Constant scores were adjusted for age and sex [29]. Specifically, we added the following values to the Constant score: male 0.15 (age − 50) if 50 ≤ age ≤ 70; 1.3 (age − 70) + 0.3 if age ≥ 70, female: 0.25 (age − 60) if 60 ≤ age ≤ 70, 0.35 (age − 70) + 2.5 if age ≥ 70. We defined nonunion as a lack of bone bridging at 1 year postoperatively, and varus progression as a varus progression of the head-shaft angle of more than 10°, based on a previous report [30]. Greater tuberosity reduction loss was defined as when the summit of the greater tuberosity was less than 5 mm below the summit of the humeral head on the anteroposterior radiograph in neutral rotation [31].

We compared the operative outcomes, postoperative reduction status, postoperative functional outcomes, and postoperative complication rates between the screw and peg groups. Furthermore, we performed a subgroup analysis to compare postoperative outcomes according to age or fracture type.

Additionally, to investigate factors influencing the occurrence of postoperative complications in this study, we compared the patient characteristics, fracture types, bone quality, calcar screw, reduction status immediately after surgery, and fixation procedures (screws or pegs) between patients who developed postoperative complications and those who did not. Bone quality was assessed using the proximal humeral cortical bone thickness measured at two levels separated by 2 cm on an anteroposterior view of the shoulder plain radiograph. In this study, the average cortical bone thickness < 6 mm was defined as local osteoporosis [32, 33].

### Statistical analysis

All statistical analyses were conducted using the SPSS software program (version 27.0; IBM Corp., Armonk, NY, USA). We used the Shapiro–Wilk test to assess the normality of continuous variables (age, body mass index [BMI], time from injury to surgery, operative time, blood loss, adjusted Constant score, ASES score, VAS, range of motion, and bone union time). We used the Student’s t-test to compare the average of variables following a normal distribution, the Mann–Whitney U-test to compare the medians of variables that did not follow a normal distribution, and Fisher’s exact test to compare the proportion of discrete variables (sex, side of injury, smoking, type of fracture, presence of local osteoporosis, reduction status, and postoperative complications). The threshold for significance was *p* < 0.05.

Reportedly, a minimal clinically important difference of the Constant score in patients with a proximal humeral fracture is 11.6 [34] and standard deviation of the Constant score in patients who underwent osteosynthesis with ALPS plate is 13.6 [23]. With a power of 0.80, a significance level of 0.05, our power analysis showed that 23 patients per group were required to show clinically significant differences of the Constant score between groups. However, based on a power analysis assuming a 42% rate of postoperative radiological complications [8], 52 patients per group would be required to show a 25% difference in complication rates.

## Results

We identified 48 patients (screw group; 25 patients, peg group; 23 patients) who met the inclusion and exclusion criteria. In this study, all patients completed follow-up at 1 year after surgery; therefore, the mean follow-up period was 1 year. The mechanism of injury was low-energy trauma in all patients. No significant differences in age, sex, side of injury, history of smoking, BMI, time from injury to surgery, presence of local osteoporosis, or fracture type were observed between the screw and peg groups (Table 1).

**Table 1:** Patient demographics

	Screw (*n* = 25)	Peg (*n* = 23)	*P*-value
Age (years)^a^	67.4 ± 14.2	73.4 ± 11.0	0.32
Sex, Male/Female^c^	11/14	6/17	0.24
Side of injury, Right/Left^c^	12/13	10/13	0.78
Smoker^c^	10	5	0.22
Body mass index^b^	23.4 (22.2–27.7)	21.5 (18.4–23.9)	0.058
Time from injury to surgery (days)^b^	7 (5–10)	5 (4–6)	0.055
Local osteoporosis^c^	12	8	0.39
Neer two-/three-part^c^	17/8	15/8	> 0.99
Medial comminution^c^	8	7	0.76
Varus displaced fracture^c^	18	14	0.54

^a^Continuous variables that followed a normal distribution are presented as the mean ± standard deviation. ^b^Continuous variables that did not follow a normal distribution are presented as the median (interquartile range). ^c^Values are presented as the number of patients

The amount of intraoperative blood loss did not differ significantly between the two groups; however, the operation time was significantly shorter in the peg group than that in the screw group (94 min vs. 114 min, *p* < 0.001, *r* = 0.52). No significant difference in the reduction status immediately after surgery was observed between the two groups (Table 2). No significant difference was observed in the number of proximal screws/pegs inserted between the two groups (9 [8–9] vs. 9 [9–9], *p* = 0.47). Even when limited to three-part fractures, no significant difference in the number of screws/pegs was observed between the two groups (9 [8–9] vs. 9 [9–9], *p* = 0.72). Calcar screw insertion (screw-calcar distance < 12 mm) [35] was confirmed in 15 patients (60%) in the screw group and 14 patients (61%) in the peg group, with no significant difference between the two groups (*p* > 0.99).

**Table 2:** Comparison of operative factors and reduction status immediately after surgery

	Screw (*n* = 25)	Peg (*n* = 23)	*P*-value
Operative time^a^ (min)	114 (98–124)	94 (83–105)	< 0.001
Blood loss^a^ (g)	130 (67–195)	84 (50–134)	0.12
Reduction status^b^			0.75
Anatomical	19	16	
Acceptable	6	7	

^a^Numbers are presented as the median (interquartile range). ^b^Values are presented as the number of patients

At 1 year postoperatively, no significant differences in the mean adjusted Constant score, the ASES score, the VAS and range of motion were observed between the two groups (Table 3).

**Table 3:** Comparison of clinical outcome scores and range of shoulder motion

	Screw (*n* = 25)	Peg (*n* = 23)	*P*-value
Adjusted Constant score	87 (82–96)	92 (82–98)	0.63
ASES shoulder score	84 (79–90)	88 (75–91)	0.81
VAS	0 (0–1.0)	0 (0–0.3)	0.41
Range of shoulder motion			
Anterior elevation (º)	120 (105–142)	140 (90–150)	0.91
Lateral elevation (º)	112 (90–135)	114 (98–130)	0.78
External rotation at sides (º)	45 (30–60)	40 (20–50)	0.28

Numbers are presented as the median (interquartile range). *ASES* American Shoulder and Elbow Surgeons, *VAS* Visual analog scale

Postoperative complications included screw/peg penetration in three patients (6.3%), avascular necrosis in one patient (2.1%), varus progression in five patients (10.4%), and greater tuberosity reduction loss in four patients (8.3%). The incidence of greater tuberosity reduction loss was significantly higher in the peg group than that in the screw group (17.4% vs. 0%, *p* = 0.046, φ = 0.32); however, no significant difference in the incidence of other complications was observed between the two groups (Table 4). Additionally, no significant difference was observed in bone union time between the screw group and the peg group (5.5 ± 1.8 months vs. 5.5 ± 1.1 months, *p* = 0.92).

**Table 4:** Comparison of postoperative complication rates

	Screw (*n* = 25)	Peg (*n* = 23)	*P*-value
Nonunion	0 (0%)	0 (0%)	> 0.99
Screw/peg penetration	1 (4.0%)	2 (8.7%)	0.60
Avascular necrosis	1 (4.0%)	0 (0%)	> 0.99
Varus progression	3 (12.0%)	2 (8.7%)	> 0.99
Greater tuberosity reduction loss	0 (0%)	4 (17.4%)	0.046

In proximal humeral fractures, patient characteristics, and fracture type distribution differ between patients ≥ 65 years and < 65 years [36]; therefore, we performed subgroup analyses of operative time, ASES score, and postoperative complications separately for patients aged 18–64 years and those aged ≥ 65 years. In the subgroup aged < 65 years, no significant differences in these outcomes were observed between the two groups. In the subgroup aged ≥ 65 years, the operative time in the peg group was significantly shorter than that in the screw group (84 min vs. 112 min, *p* = 0.001, *r* = 0.54) (Supplementary Table 1).

Additionally, we performed subgroup analyses to compare the outcomes in Neer type II and type III fractures. In Neer type II fractures, the incidence of greater tuberosity reduction loss was significantly higher in the peg group than in the screw group (33.3% vs. 0%, *p* = 0.047, φ = 0.45); however, no significant differences were observed in other complications or in adjusted Constant and ASES scores. In type III fractures, no significant difference was observed in postoperative outcomes between the two groups (Supplementary Table 2).

Patients who developed postoperative complications had significantly lower adjusted Constant and ASES scores compared to those who did not. However, no significant differences were observed between the two groups in patient characteristics, fracture types, reduction status immediately after surgery, or fixation procedures (Supplementary Table 3).

Among these complications, patients who experienced postoperative greater tuberosity reduction loss had significantly lower adjusted Constant and ASES scores compared to those who did not. Neer three-part fractures and the use of proximal pegs were significantly associated with postoperative greater tuberosity reduction loss (Table 5).

**Table 5:** Risk factors of greater tuberosity reduction loss

	No greater tuberosity reduction loss (*n* = 44)	Greater tuberosity reduction loss (*n* = 4)	*P*-value
Age (years)^a^	74.0 (68.0 − 78.9)	80.5 (59.0–87.8)	0.19
Sex, Male/Female^b^	17/27	0/4	0.28
Side of injury, Right/Left^b^	20/24	2/2	> 0.99
Smoker^b^	14	1	> 0.99
Body mass index^a^	23.3 (20.7–27.0)	20.1 (18.4–22.8)	0.17
Time from injury to surgery (days)^a^	5 (4 − 8)	5 (3–8)	0.48
Local osteoporosis^b^	18	2	> 0.99
Neer two-/three-part^b^	31/13	0/4	0.012
Medial comminution^b^	14	1	> 0.99
Varus displaced fracture^b^	30	1	0.12
Reduction status, Anatomical/Acceptable^b^	33/11	2/2	0.29
Fixation procedure, Screw/Peg^b^	25/19	0/4	0.045
Adjusted Constant score	92 (82–98)	75 (59–86)	0.039
ASES score	87 (78–97)	67 (60–76)	0.005

^a^Continuous variables that did not follow a normal distribution are presented as the median (interquartile range). ^b^Values are presented as the number of patients. *ASES* American Shoulder and Elbow Surgeons

## Discussion

In this study, we compared the functional and radiological outcomes of osteosynthesis using anatomical locking plates with the use of proximal screws and with the use of proximal pegs for proximal humeral fractures. The functional outcomes obtained in this study were comparable with those reported in the previous study [6]. However, the incidence of postoperative radiological complications was slightly lower than that reported in previous studies [6–8], which may be explained by the exclusion of four-part fractures and fracture types associated with a high risk of avascular necrosis in this study. We made three important clinical observations.

First, this study showed no significant difference in postoperative shoulder functional outcomes between the two groups. Our results concur with the findings of the previous studies that the postoperative functional outcomes or range of motion were not significantly different in plate fixation with proximal screws and plate fixation with smooth pegs [21–23]. The use of smooth pegs was expected to decrease the risk of peg penetration; however, our study revealed no significant difference in the frequency of screw or peg penetration between the two groups, which is also in line with the previous reports [21–23]. The frequency of postoperative varus progression was also not significantly different between the two groups in this study. This finding is similar to previous clinical studies [21–23] and supported by the biomechanical studies [18, 19] that reported no difference in biomechanical characteristics of plate fixation for proximal humeral fracture model when proximal screws were compared with proximal pegs.

Second, postoperative displacement of the greater tuberosity fragment was significantly higher in the peg group than that in the screw group. A previous clinical study also showed that loss reduction of the greater tuberosity tended to be more frequent in the peg group than in the screw group, although the difference was not significant [21]. These findings may be partly explained by the difference in fixation strength between threaded screws and smooth pegs for small and comminuted greater tuberosity fragments. A previous study using finite element models of two-part fractures reported that threading the smooth peg increased the varus bending stiffness of proximal humeral plate constructs [20]. However, the previous biomechanical studies comparing proximal screws and pegs have not involved a three-part fracture with greater tuberosity fragments [18–20]. In a biomechanical study of distal radius fractures [37], fixation of small lunate fragments was stronger with threaded screws compared with smooth pegs, suggesting that threaded screws may be stronger for small bone fragments. Alternatively, the design of the ALPS plate to avoid postoperative subacromial impingement resulted in less buttressing coverage of the greater tuberosity, which may reduce the fixation of the greater tuberosity fragments [21]. However, owing to the limited number of cases with available postoperative CT, we could not compare the buttressing coverage of the two plates radiologically in this study; further research is needed. The fixation strength may also depend on the diameter of the core of the proximal screws and the number of screws. A previous study using a two-part osteoporotic proximal humeral fracture model reported that screws with larger core diameters increased the stability of the locking plate [38]. However, in this study, the peg group, which used proximal implants with larger core diameters, had a higher incidence of greater tuberosity reduction loss. This discrepancy suggests that the threading may have a greater influence on the fixation of the greater tuberosity fragments than the core diameter of the screw/peg. Another previous study using a three-part proximal humeral fracture model reported that the number of proximal screws contributes to biomechanical stability [39]. However, in this study, no significant difference in the number of screws/pegs was observed between the two groups, suggesting that the number of screws or pegs did not influence the difference in the incidence of greater tuberosity reduction loss.

Third, our study revealed that the operative time was significantly shorter in the peg group than that in the screw group, especially in the older patients. This finding may be related to the difference in time required to insert between 7 and 10 screws and pegs. In addition, the ALPS plate has a drill sleeve attached to the screw/peg holes of the plate, whereas the MODE^®^ plate requires attachment and removal of the drill sleeve for each screw insertion, which may explain the difference in operative time. Despite these hypotheses, the mechanism remains unclear because this study did not evaluate the operative time required for each step during surgery in detail. However, our study suggests that the use of smooth pegs for fixation of the humeral head may reduce the operative time.

Postoperative radiological complications have been associated with various risk factors. Older age, female sex, and absence of medial comminution have been proposed as risk factors for postoperative fixation failure [10, 40]; however, another study [12] found no significant association between these factors and postoperative complications, which is consistent with our results. Although poor bone quality was also associated with radiological complications [10], no association was found between local osteoporosis and complications in this study. Our results are consistent with a previous study that reported a significant association between poor bone quality and radiological complications only in intramedullary nailing, not in plate fixation [33]. Neer four-part fractures and postoperative malreduction were also related to postoperative screw perforation or poor outcomes [12, 17, 26]; however, no cases with these factors were included in this study, and therefore they were not identified as risk factors. When limited to reduction loss of the greater tuberosity, the use of pegs and three-part fractures were identified as risk factors in this study. In this study, as in previous studies [31, 35, 41], reduction loss of the greater tuberosity was associated with poor functional outcomes; therefore, the use of proximal screws or plates that adequately cover the greater tuberosity is recommended for three-part fractures involving this region.

The strength of this study was that all surgeries were performed by the same surgeon using the same surgical approach, thus minimizing the influence of differences in surgeons and surgical techniques. In addition, previous reports on the postoperative outcomes of the ALPS plate have used high and low plates depending on the fracture type and a mixture of screws and pegs for fixation of the proximal bone fragments [21, 23]. In contrast, in this study, high plates using proximal smooth pegs only were used in all surgeries in the peg group, reflecting outcomes of unified implants.

However, this study had some limitations. First, this is a retrospective cohort study, not a randomized prospective study. Although not statistically significant, the tendency toward older age and higher BMI in the screw group may represent a hidden bias that could affect the outcome. In addition, unobserved differences may have biased the results. For example, although the assistants in this study had similar surgical experience, differences in their skills between the two groups may have affected the outcomes. Additionally, the surgical instruments and fluoroscopy devices used in this study were mostly uniform; however, institutional variability between the two hospitals may have introduced bias. Furthermore, the study design, which categorized patients into two groups based on the period during which surgery was performed, may have introduced selection and time-dependent bias. Although no significant differences in postoperative functional outcomes and reduction status were observed between the two groups, the shorter operative time in the peg group may be attributable to the surgeon’s increasing experience over time. Second, both the ALPS plate and the MODE^®^ plate are anatomical plates designed for the humerus anatomy; however, the detailed geometry of the plates is different. Therefore, use of the same plate would more accurately evaluate the effect of proximal screws versus pegs on surgical outcomes. However, in the previous studies comparing the outcomes of plate fixation with proximal screws and smooth pegs, the PHILOS^®^ plate (Synthes, Oberdorf, Switzerland) was used in the screw group and the ALPS plate was used in the peg group [21–23]. The PHILOS plate is a non-anatomical locking plate that does not distinguish between left and right sides. This study is the first to use anatomical plates in the screw group. Third, although functional and radiological outcomes were evaluated at 1 year postoperatively, longer-term outcomes were not assessed. Fourth, the cohort of patients available for analysis was relatively small. The power analysis suggests that the results of the incidence of radiological complications may be affected by undetected factors (β-error). In particular, the differences in screw/peg penetration between the two groups may not have been detected due to the small number of cases and the low incidence of this complication. Additionally, the significant difference in greater tuberosity reduction loss may be subject to statistical fragility because only four patients in this study developed this complication. Fifth, all surgeries were performed by a single surgeon, which may have resulted in potential surgeon bias and reduced the generalizability of the outcomes.

## Conclusions

This study provides new information on the effect of the use of proximal smooth pegs on the postoperative outcomes of osteosynthesis using an anatomical plate for proximal humeral fractures. Our study showed that the use of proximal pegs did not significantly affect postoperative shoulder functional outcomes, although it may reduce operative times. In three-part fractures involving the greater tuberosity, the use of proximal screws or plates with sufficient coverage of the greater tuberosity may be desirable to prevent reduction loss of the greater tuberosity.

## Supplementary Information

Supplementary Material 1.

Supplementary Material 2.

Supplementary Material 3.

## Data Availability

Data supporting this study’s findings are available from the corresponding author on reasonable request.

## Abbreviations

ASES: American Shoulder and Elbow Surgeons
VAS: Visual analog scale
BMI: Body mass index

## Declarations

This retrospective study was performed according to the Declaration of Helsinki. Opt-out consent relies on implicit consent, where willingness to participate is tacit or presumed and can be retracted by active objections.
