# Supplementary material for: Comparison of surgical outcomes of osteosynthesis using anatomical locking plates with proximal screws and smooth pegs for proximal humeral fractures
Source: BMC Musculoskelet Disord. 2025 Jul 9;26:668. doi: 10.1186/s12891-025-08917-0 (PMC12239504; doi:10.1186/s12891-025-08917-0)
Supplement: Supplementary file 1 — Supplementary Material 1. [file 12891_2025_8917_MOESM1_ESM.docx]

**Supplementary Table 1. Subgroup analysis of Postoperative Outcomes in patients < 65 years and ≥ 65 years**

| < 65 years | Screw (n=8) | Peg (n=6) | P-value |
| --- | --- | --- | --- |
| Operative time (min) * | 119 (103–124) | 108 (98–115) | 0.18 |
| ASES shoulder score * | 89 (83–95) | 81 (79–91) | 0.85 |
| Screw/peg penetration † | 0 (0%) | 0 (0%) | >0.99 |
| Avascular necrosis † | 1 (12.5%) | 0 (0%) | >0.99 |
| Varus progression † | 1 (12.5%) | 1 (16.6%) | >0.99 |
| Greater tuberosity reduction loss † | 0 (0%) | 1 (16.6%) | 0.43 |

| ≥ 65 years | Screw (n=17) | Peg (n=17) | P-value |
| --- | --- | --- | --- |
| Operative time (min) * | 112 (97–122) | 84 (79–96) | 0.001 |
| ASES shoulder score * | 82 (78–90) | 81 (69–92) | 0.68 |
| Screw/peg penetration † | 1 (5.9%) | 2 (11.8%) | >0.99 |
| Avascular necrosis † | 0 (0%) | 0 (0%) | >0.99 |
| Varus progression † | 2 (11.8%) | 1 (5.9%) | >0.99 |
| Greater tuberosity reduction loss † | 0 (0%) | 3 (17.6%) | 0.23 |

* Numbers are presented as the median (interquartile range). † Values are presented as the number of patients. ASES = American Shoulder and Elbow Surgeons.
