# Supplementary material for: Comparison of surgical outcomes of osteosynthesis using anatomical locking plates with proximal screws and smooth pegs for proximal humeral fractures
Source: BMC Musculoskelet Disord. 2025 Jul 9;26:668. doi: 10.1186/s12891-025-08917-0 (PMC12239504; doi:10.1186/s12891-025-08917-0)
Supplement: Supplementary file 2 — Supplementary Material 2. [file 12891_2025_8917_MOESM2_ESM.docx]

**Supplementary Table 2. Subgroup analysis of Postoperative Outcomes in Neer type II and III fractures**

| Neer type II fractures | Screw (n=12) | Peg (n=12) | P-value |
| --- | --- | --- | --- |
| Adjusted Constant score * | 86 (81–96) | 86 (78–98) | 0.93 |
| ASES shoulder score * | 82 (79–89) | 84 (64–90) | 0.80 |
| Screw/peg penetration † | 0 (0%) | 1 (8.3%) | >0.99 |
| Avascular necrosis † | 1 (8.3%) | 0 (0%) | >0.99 |
| Varus progression † | 1 (8.3%) | 1 (8.3%) | >0.99 |
| Greater tuberosity reduction loss † | 0 (0%) | 4 (33.3%) | 0.047 |

| Neer type III fractures | Screw (n=13) | Peg (n=11) | P-value |
| --- | --- | --- | --- |
| Adjusted Constant score * | 88 (80–97) | 93 (84–100) | 0.46 |
| ASES shoulder score * | 88 (78–92) | 88 (76–92) | 0.87 |
| Screw/peg penetration † | 1 (7.7%) | 1 (9.1%) | >0.99 |
| Avascular necrosis † | 0 (0%) | 0 (0%) | >0.99 |
| Varus progression † | 2 (15.4%) | 1 (9.1%) | >0.99 |
| Greater tuberosity reduction loss † | 0 (0%) | 0 (0%) | >0.99 |

* Numbers are presented as the median (interquartile range). † Values are presented as the number of patients. ASES = American Shoulder and Elbow Surgeons.
