# Supplementary material for: Comparison of surgical outcomes of osteosynthesis using anatomical locking plates with proximal screws and smooth pegs for proximal humeral fractures
Source: BMC Musculoskelet Disord. 2025 Jul 9;26:668. doi: 10.1186/s12891-025-08917-0 (PMC12239504; doi:10.1186/s12891-025-08917-0)
Supplement: Supplementary file 3 — Supplementary Material 3. [file 12891_2025_8917_MOESM3_ESM.docx]

**Supplementary Table 3. Risk factors of Postoperative Complications**

|  | No complication (n=39) | Complication (n=9) | P-value |
| --- | --- | --- | --- |
| Age (years) * | 69.0 ± 14.1 | 73.4 ± 16.7 | 0.88 |
| Sex, Male/Female † | 14/25 | 3/6 | >0.99 |
| Side of injury, Right/Left † | 16/23 | 6/3 | 0.27 |
| Smoker † | 12 | 3 | >0.99 |
| Body mass index ** | 23.2 (20.0-25.9) | 23.6 (20.1-27.9) | 0.57 |
| Time from injury to surgery (days) ** | 5 (5-9) | 4 (3-7) | 0.17 |
| Local osteoporosis † | 16 | 4 | >0.99 |
| Neer two-/three-part † | 27/12 | 4/5 | 0.25 |
| Medial comminution † | 14 | 1 | 0.24 |
| Varus displaced fracture † | 27 | 4 | 0.25 |
| Reduction status, Anatomical/ Acceptable † | 27/10 | 6/3 | 0.70 |
| Fixation procedure, Screw/Peg† | 21/18 | 4/5 | 0.70 |
| Adjusted Constant score ** | 92 (84-98) | 82 (72-87) | 0.027 |
| ASES score ** | 88 (79-92) | 78 (67-83) | 0.006 |

* Continuous variables that follow a normal distribution are presented as the mean ± standard deviation. ** Continuous variables that did not follow a normal distribution are presented as the median (interquartile range). † Values are presented as the number of patients. ASES = American Shoulder and Elbow Surgeons.
